# Effects of GnRHa treatment during vitellogenesis on the reproductive physiology of thermally challenged female Atlantic salmon (*Salmo salar*)

**DOI:** 10.7717/peerj.3898

**Published:** 2017-10-20

**Authors:** Kelli Anderson, Ned Pankhurst, Harry King, Abigail Elizur

**Affiliations:** 1 Genecology Research Centre, Faculty of Science, Health, Education and Engineering, University of the Sunshine Coast, Sippy Downs, Queensland, Australia; 2 Australian Seafood Cooperative Research Centre, Bedford Park, South Australia, Australia; 3 Australian Rivers Institute, Griffith University, Gold Coast, Queensland, Australia; 4 Salmon Enterprises of Tasmania, Wayatinah, Tasmania, Australia

**Keywords:** Temperature, Atlantic salmon, Fertility, Gene expression, Reproduction, Gonadotropin releasing hormone

## Abstract

Tasmanian Atlantic salmon (*S. salar*) broodstock can experience temperatures above 20 °C, which impairs reproductive development and inhibits ovulation. The present study investigated the prolonged use of gonadotropin releasing hormone analogue (GnRHa) during vitellogenesis as a means of maintaining endocrine function and promoting egg quality at elevated temperature in maiden and repeat spawning *S. salar*. GnRHa-treatment during vitellogenesis did not compensate for the negative effects of thermal challenge on the timing of ovulation, egg size, egg fertility or embryo survival in any fish maintained at 22 °C relative to 14 °C. The lack of effectiveness was reflected by the endocrine data, as plasma follicle stimulating hormone and luteinising hormone levels were not different between treated and untreated groups at 22 °C. Furthermore, plasma testosterone and E2 levels were unchanged in GnRHa-treated fish at 22 °C, and plasma levels were generally lower in both groups maintained at 22 °C relative to 14 °C. Transcription of vitellogenin, and zona pellucida B and C was not enhanced in GnRHa-treated fish relative to untreated fish at 22 °C, presumably due to observed suppression of plasma E2. These results indicate that thermal impairment of reproduction is likely to occur on multiple levels, and is difficult to overcome via hormonal manipulation.

## Introduction

Increases in ambient water temperature can fundamentally affect the physiology of farmed Atlantic salmon, *Salmo salar* (Battaglene et al., 2008), which is especially relevant for Tasmanian *S. salar* that are already reared towards their upper-limit of thermal tolerance (Pankhurst & King, 2010). As a result of exposure to elevated temperature, a marked decrease in circulating plasma 17β-estradiol (E2) typically occurs in females (King, Pankhurst & Watts, 2007; Pankhurst et al., 2011), which has the downstream effect of reducing vitellogenesis and zonagenesis (Pankhurst et al., 2011). The molecular basis for reduced E2 synthesis during peak vitellogenesis likely occurs at multiple levels, with reductions in the expression of ovarian steroidogenic enzymes involved in the biosynthesis of both testosterone (T) and E2 (Anderson et al., 2017; Anderson & Elizur, 2012). Downstream of E2 synthesis, there is evidence to suggest that hepatic E2 receptor (Er) binding affinity is reduced at higher temperatures (Watts et al., 2005); however, the expression of *er* is not modulated by temperatures of up to 22 °C in adult female Tasmanian *S. salar* (Anderson & Elizur, 2012). This suggests that the physiological effects of reduced E2 tone may have been exacerbated by decreased efficiency of E2 receptor binding.

The collective effect of suppressed endocrine dysfunction at high temperature in *S. salar* and other salmonids such as rainbow trout (*Oncorhynchus mykiss*) is a reduction in egg fertility and embryo survival (King et al., 2003; Pankhurst et al., 1996; Taranger & Hansen, 1993). However, a study by Pankhurst et al. (2011) showed that egg quality was reduced to greater extent in first-time (maidens, 2+ years old) than in second-time (repeats, 3+ years old) spawning *S. salar* reared at 22 °C compared to their respective controls at 14 °C. As such, the use of repeat spawning fish as opposed to maidens may partially offset the negative impacts of elevated temperature on egg quality in Tasmanian *S. salar*. However, it is not viewed as a viable option due to the higher cost and risk associated with rearing large fish intensively for an additional year. Alternatively, hormonal therapy may maintain endocrine function and subsequent egg quality through critical periods at elevated temperature. However, the potential of such management strategies remain to be explored.

Gonadotropin-releasing hormone (GnRH) has been used since the 1970s to synchronise and induce ovulation by stimulating the secretion of endogenous luteinizing hormone (Lh) in a wide range of species (Zohar & Mylonas, 2001). Its effectiveness has also been tested at 16 °C in *S. salar*, with GnRH analogue (GnRHa) treatment in combination with a temperature ramp down to 8 °C advancing ovulation and maintaining egg fertility relative to sham treated fish (King & Pankhurst, 2004b). In addition to the traditional role of inducing final oocyte maturation (FOM), treatment with GnRHa also resulted in an increase in follicle stimulating hormone beta (*fshβ*) gene expression, and a subsequent increase in culture medium Fsh levels in a coho salmon (*Oncorhynchus kisutch*) pituitary cell culture (Dickey & Swanson, 2000). In female *S. salar*, *in vivo* GnRHa implantation can result in a premature rise in plasma E2 and/or T (King & Pankhurst, 2007; Crim, Glebe & Scott, 1986), and promote egg development relative to untreated fish (Crim, Glebe & Scott, 1986). The Atlantic and coho salmon in the those studies were vitellogenic at the time of GnRH-treatment, which demonstrates pituitary responsiveness to GnRH during the earlier stages of reproductive development in terms of Fsh production.

Little information is available on the effects of prolonged GnRH-treatment on the endocrine system and oocyte development during vitellogenesis. However, in non-salmonids, GnRH is capable of stimulating the complete cycle of oocyte development, growth and maturation (Morehead, Pankhurst & Ritar, 1998), and in salmonids, it appears as though GnRH treatment is able to simulate the production and release of Fsh during vitellogenesis. Since there is no evidence to suggest that the expression of ovarian follicle stimulating hormone receptor (*fshr*) is reduced at elevated temperature in *S. salar* (Anderson et al., 2017), as it is in other species such as pejerrey (*Odontesthes bonariensis*) (Soria, Strüssmann & Miranda, 2008), it is possible that treatment with GnRH will enhance endocrine function in female *S. salar* broodstock during vitellogenesis. Thus, the aim of the present study was to investigate whether prolonged GnRH therapy during reproductive development could offset the inhibitory effects of thermal challenge in terms of endocrine function and egg quality in maiden and/or repeat spawning *S. salar* broodstock. To do this, the effects of implantation with GnRH on plasma levels of pituitary hormones, gonadal steroids and Vtg, as well as E2-dependent hepatic gene expression of *vtg*, zona pellucida B (*zpb*) and C (*zpc*) were determined in maiden and repeat spawning fish throughout reproductive development.

## Methods

### Fish husbandry and maintenance

**Figure 1:**
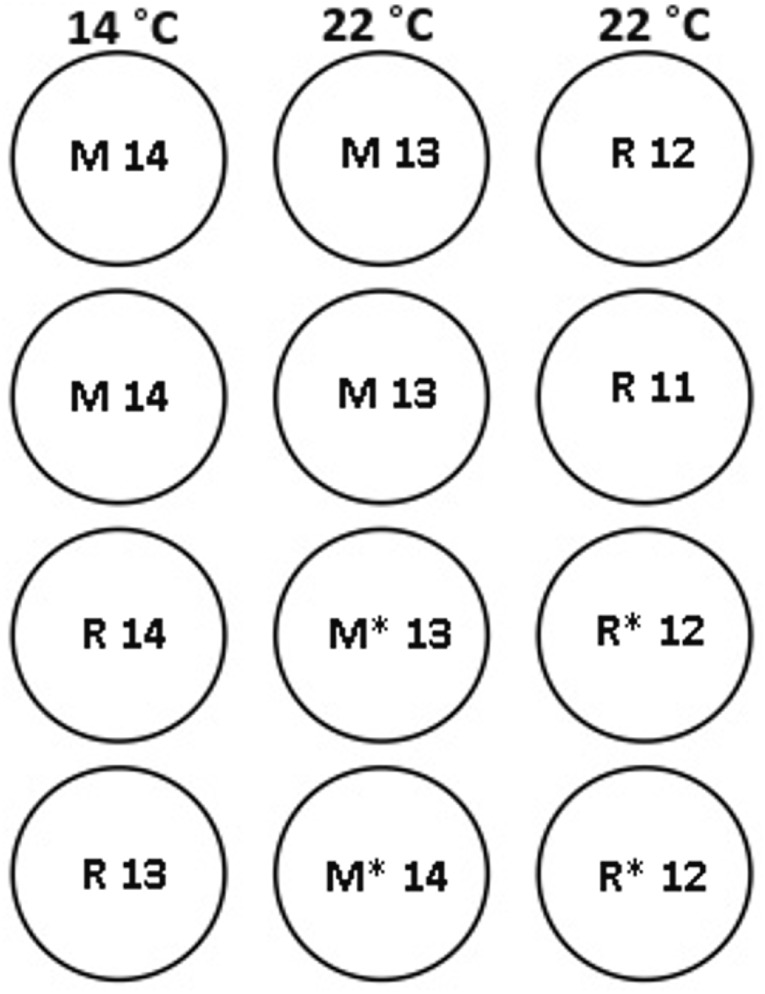
Experimental tank layout for thermal and hormonal manipulations from January 22nd. M and R represent maiden and repeat-spawning fish respectively, GnRH administration is marked by an asterisk, lack of an asterisk denotes groups that did not receive an implant, and the number of fish per tank is also indicated.

**Figure 2:**
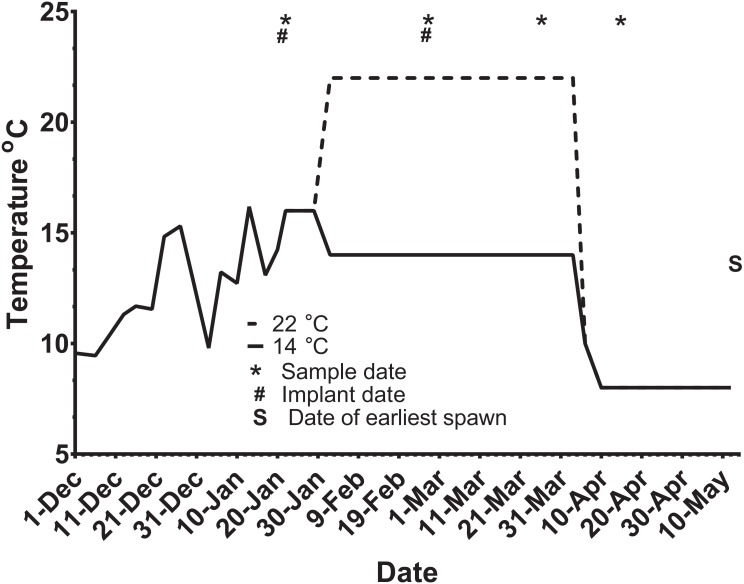
Thermal adjustment and hormone implantation timeline for untreated and GnRH-treated maiden and repeat spawning female Atlantic salmon broodstock maintained at 14 or 22 °C during vitellogenesis. From October to January 22nd fish were exposed to ambient photoperiod and temperature, thereafter temperature was maintained at either 14 °C or 22 °C. Average ambient water temperatures for October and November were 9.3 and 11.6 °C, respectively. Sampling and implantation dates, and the date of earliest spawning, are indicated.

Ninety-five maiden and 88 repeat adult females from the Salmon Enterprises of Tasmania (Saltas) spawning stock were held in 200 (maidens) or 50 (repeats) m^3^ circular tanks at ambient photoperiod and temperature under standard conditions of husbandry at the Saltas Wayatinah hatchery until January 2009. In mid-January, fish were transferred to temperature-controlled 4 m^3^ Rathbun tanks (maximum 14 fish per tank, numbers varied according to the availability of stock) under simulated ambient photoperiod according to the following 6 treatment groups; maiden 14 °C, repeat 14 °C, maiden 22 °C, maiden GnRH implant 22 °C, repeat 22 °C and repeat GnRH implant 22 °C (Fig. 1). At the time of transfer, tank temperature was set to match ambient water temperature, and the experimental temperatures were then reached by adjustment a rate not exceeding 1 °C per day. Fourteen and 22 °C represent typical cool, and warm Tasmanian summers respectively. Fish were not fed from the time of transfer to the temperature controlled systems in January consistent with hatchery practice for management of this experimental stock of fish. All fish were maintained at the nominated temperature (14 or 22 °C) until late March when all fish were exposed to a gradual temperature ramp down to 8 °C (Fig. 2) to induce final oocyte maturation and ovulation as in King & Pankhurst (2000).

### Sampling protocol

At each destructive sample point (all except the last date), six or seven fish were sampled for each treatment, and fish were selected at random from each replicate tank to ensure that stocking density was comparable between tanks. Maiden and repeat fish reared at ambient temperature were sampled in late October 2008 to establish an early-vitellogenic baseline for each group, and on the 22nd January 2009 at the same time that temperature transfer to 14 or 22 °C occurred for the remaining fish (Fig. 2). Subsequent samples were taken from each group during February 25–27, March 26–27, and April 15–16. After the last destructive sampling in April, five (repeat 22 °C GnRH-treated), six (maiden 22 °C, repeat 22 °C, and repeat 14 °C), or seven fish (maiden 14 °C and maiden 22 °C GnRH-treated) were left to proceed through to ovulation and stripping. A subset of maidens at 22 °C and repeats at 22 °C were implanted with an Ovaplant™ (Syndel, Nanaimo, Canada) cholesterol pellet containing 37 µg of the D-Arg^6^, Pro^9^NEt (GnRHa) on 22nd January (during temperature transfer), and again over February 26–27 to give repeat doses of approximately 7–12 µg kg^−1^. The dose chosen was designed to stimulate or maintain pituitary secretion of Fsh, but remain below the threshold likely to stimulate an Lh surge and possible premature stimulation of oocyte maturation and ovulation (King & Pankhurst, 2004a). Untreated fish did not receive a sham implant, to simulate real industry conditions.

For sampling, fish were netted from the holding tanks, terminally anaesthetised in Aqui-S™ (Crop and Food, New Zealand), weighed, measured and then blood sampled by caudal puncture using pre-heparinised syringes. Blood plasma was centrifuged at 12,000 g, and stored frozen at −20 °C for later measurement of plasma hormones. Ovaries were excised, weighed and portions allocated to 50-ml pots containing teleost saline for fecundity estimation and follicle measurement. Segments of liver were transferred to 1–2 ml of RNA Later™ (Qiagen, Germany), kept at 4 °C overnight then stored at −80 °C.

Gonadosomatic index (GSI) was calculated as (gonad weight/total body weight) ×100, and condition factor (CF) as (body weight/length^3^) ×100. Fecundity was determined for a 5 g ovarian segment, and total fecundity was determined by correction for total ovarian weight and expressed as relative fecundity kg^−1^ body weight as in Pankhurst et al. (2011). Using the procedure described in King & Pankhurst (2004a), fish were checked for ovulation by applying gentle pressure to the abdomen, and if ovulation had occurred, ova were stripped and fertilised for measurement of egg size, fertility and survival to the eyed stage at 250 degree-days of incubation. All animal experiments were conducted in accordance with Australian law under ethical approval issued by the Griffith University and University of the Sunshine Coast Animal Ethics Committees (ENV/25/08AEC and AN/A/09/44 respectively).

### Plasma hormone and vitellogenin measurement

Plasma Fsh (all months) and Lh (March and April only) measurements were performed using an RIA developed for *O. kisutch* by Swanson et al. (1989) with the reagents and modifications described in Anderson & Elizur (2012). The cross reactivity of Fsh in the Lh assay and Lh in the Fsh assay were approximately 4.4% and 6%, respectively (Swanson et al., 1989). ANCOVA was performed as part of a previous study which confirmed that parallelism was present between serial dilutions of *S. salar* plasma and the purified coho standards (Anderson & Elizur, 2012). The limit of detection (LOD) of the Fsh and Lh assays was approximately 0.6 and 0.5 ng ml^−1^ respectively.

Plasma levels of E2, T and cortisol (F) were determined by radioimmunoassay using the reagents and procedure for E2 and T described in Pankhurst & Carragher (1992), and for F as in Pankhurst et al. (2008) from 100 µl of plasma extracted with 1 ml ethyl acetate. Extraction efficiency was 78%, 79% and 74% for E2, T and F respectively as determined by recovery of ^3^H-labelled steroid from replicates of a plasma pool. Assay values were corrected accordingly to account for extraction losses. Interassay variability was determined by repeat measurement of a pooled internal standard and was (CV%) 7.4 (*n* = 3), 13.9 and 12.3 (*n* = 2) for E2, T and F respectively.

Plasma Vtg levels were measured by enzyme linked immunosorbent assay using the reagents and protocol as described in Watts et al. (2003). Interassay variability was assessed by repeat measurement of a Vtg standard from the central part of the assay curve and was 13.1 (CV%, *n* = 7). Pooled internal standards were not used here due to the tendency of Vtg to denature following repeated freeze-thaw cycles. The lower LOD for the assay was 80 ng ml^−1^.

### RNA extraction and cDNA synthesis

Total RNA was isolated from 15 mg of hepatic tissue using the Illustra RNAspin Mini kit (GE Healthcare, Little Chalfont, UK) according to the manufacturer’s protocol. RNA yield and 260/280 purity ratio were determined using the NanoDrop 2000 (Thermo Scientific, Waltham, MA, USA). An RNA integrity number (RIN) was determined for a random sample of hepatic RNA (*n* = 48) using a 2,100 bioanalyzer (Agilent, Santa Clara, CA, USA) to establish RNA quality. Four hundred nanograms of liver-derived RNA were then used to synthesise cDNA for use in real-time quantitative PCR (qPCR) using the QuantiTect^®^ reverse transcription kit (Qiagen, Hilden, Germany). This kit includes a DNA elimination step to remove potential contamination of qPCRs by genomic DNA.

### Hepatic gene expression

Gene specific primers (Gsps) for target genes previously optimised and validated for qPCR (Pankhurst et al., 2011) were used to amplify the target hepatic transcripts *vtg*, *zpc* and *zpb* (primers detect both *zpba* and *zpbb*, collectively referred to as *zpb*, Table 1), and the reference transcripts TATA binding protein (*tbp*), hypoxanthine phosphoribosyltransferase 1 (*hprt1*), beta-tubulin (*β*-*tub*) and elongation factor 1 alpha (*ef1α*, Table 1). The suitability of using candidate reference genes for normalisation was assessed using GeNorm (Vandesompele et al., 2002) and BestKeeper (Pfaffl et al., 2004) through the site RefFinder (Xie et al., 2012) (Table S1), and the output from these algorithms was then independently validated as suggested by Anderson & Elizur (2012) and Setiawan & Lokman (2010). As a result of this analysis, *tbp* was used for accurate target gene normalisation, and the level of target gene expression relative to a calibrator sample (pool of randomly selected hepatic cDNA) analysed in every run was calculated using Rest©2008 V2.0.7 (Pfaffl, Horgan & Dempfle, 2002).

**Table 1:** qPCR Gsps.

Gene name		Sequence (5′ → 3′)	Prod. size, bp	*E*	Source sequence
Vtg	F	AAC TTT GCC CCT GAA TTT GC	95	0.984	DQ834857
	R	GCT CTA GCC AGA CCC TCC GC			
Zpb	F	GTT TCC AGG GAT GCC ACT CT	113	0.937	AJ000664, AJ000665
	R	TGG TAG ATG GCA AAG GCA GA			
Zpc	F	GTC CCC CTG CGT ATC TTT GT	121	0.969	GU075906
	R	AAC CTG TCA CTT TGG CAT CG			
Hprt1	F	GAT GAT GAG CAG GGA TATGAC	165	0.963	BT043501
	R	GCA GAG AGC CAC GAT ATG G			
Tbp	F	TCC CCA ACC TGT GAC GAA CA	117	0.981	BT059217
	R	GTC TGT CCT GAG CCC CCT GA			
Ef1α	F	GCA CCA CGA GAC CCT GGA AT	94	0.969	AF321836
	R	CAC GTT GCC ACG ACG GAT AT			
β-tubulin	F	CCG TGC TTG TCG ACT TGG AG	144	0.975	DQ367888
	R	CAG CGC CCT CTG TGT AGT GG			

**Notes.**

E efficiency bp base pairs

**Figure 3:**
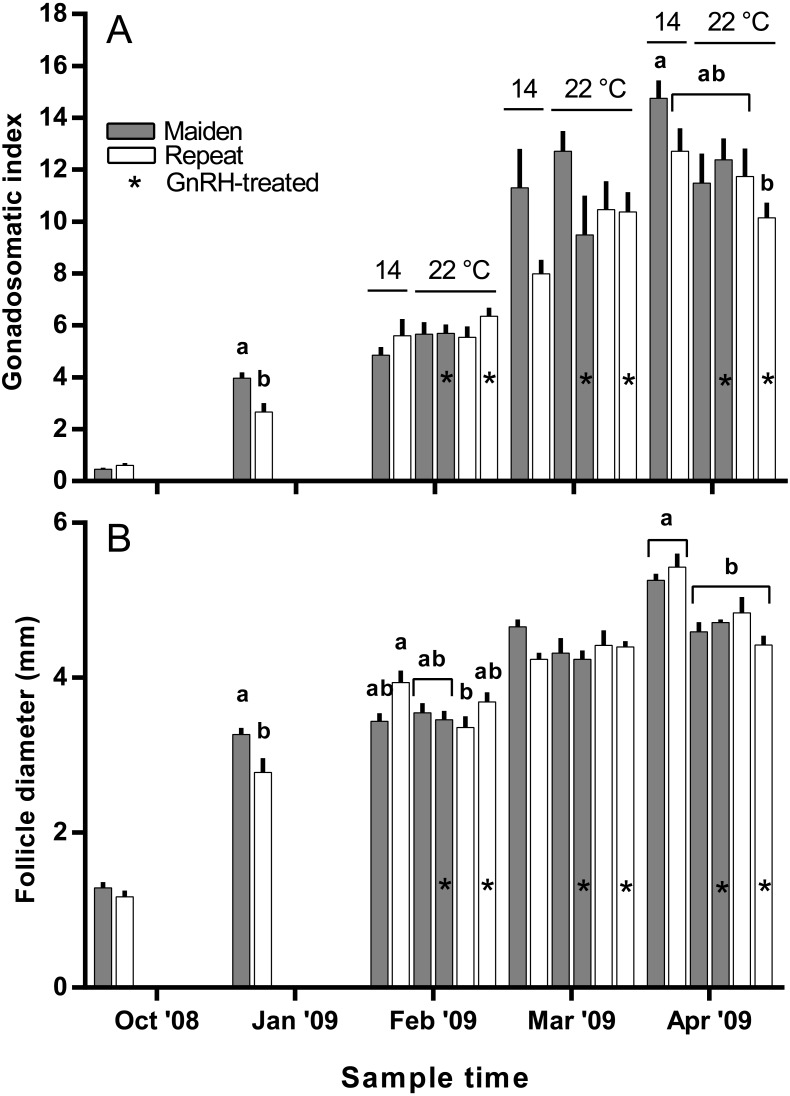
Mean + SEM (*n* = 6–7) gonadosomatic index (A) and follicle diameter (B) of maiden and repeat spawners. Different superscripts among sample times denote significant differences (*p* ≤ 0.05). Other details as for Fig. 2.

qPCRs were conducted on a Rotor-gene 6,000 series thermal cycler (Qiagen, Hilden, Germany) using the Platinum^^®^^ SYBR^^®^^ Green qPCR SuperMix-UDG (Invitrogen, Carlsbad, CA, USA) master mix and the following cycling conditions: 50 °C for 2 min; 95 °C for 2 min; 40 cycles of 95 °C for 15 s; 60 °C for 15 s, and 72 °C for 20 s (acquiring). At the end of cycle 40, a melt curve analysis was performed to confirm amplification of a single product as follows: 90 s preconditioning step at 72 °C, followed by a temperature gradient up to 95 °C at 1 °C per 5 s. The 10 µl qPCR reaction contained 5 µl SYBR, 200 nM each primer, 3.6 µl PCR grade water and 1 µl cDNA template. For every gene analysed no-template controls and a calibrator were included to detect possible contamination, and control for in-between run variability, respectively. Negative reverse transcription controls were also analysed to detect the presence of any contaminating genomic DNA.

### Statistical analysis

Pairwise comparison of means of morphometric and plasma hormone data was made using an independent samples *t*-test (October and January). For multiple comparisons (following months) one-way ANOVA with post-hoc comparison of means by Tukeys-b was performed using the SPSS (version 15.0) statistical package. Differences in relative gene expression levels were detected non-parametrically using the Kruskal–Wallis test coupled with Bonferroni’s Correction to reduce the risk of type 1 error. The *P* value for significance was set at 0.05 for all analyses.

## Results

### Morphometric data

**Figure 4:**
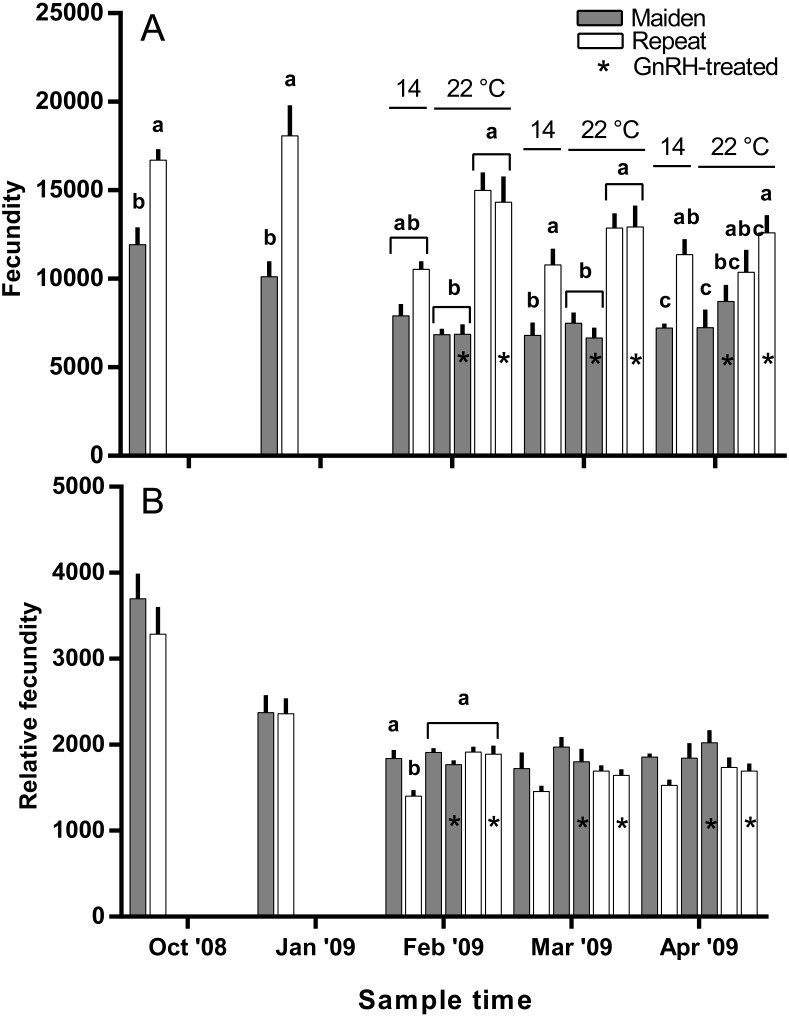
Mean + SEM (*n* = 6–7) absolute (A) and relative fecundity (B) of maiden and repeat spawners. Other details as for Fig. 2.

The mean (±SEM) body weight of maiden spawners (4.01 ± 0.07 kg) was consistently lower than that of repeat spawners (7.03 ± 0.15 kg) during the course of the experiment (data not shown). CF was lower in repeats than maidens in October; however, CF was not different among fish from all groups thereafter (data not shown). There was a general trend of increasing GSI and follicle diameter with sample time across all treatment groups (Figs. 3A and 3B). The GSI of fish from all treatments was similar after introduction to the temperature-controlled tanks and hormonal treatment; however, follicle diameter was lower in all fish reared at 22 °C relative to their respective 14 °C control in April, and did not change as a result of treatment with GnRH. Total fecundity (during development) was highest in repeat spawners, but there was no consistent difference in relative fecundity between repeats and maidens, and no discernible effect of temperature regime or hormonal treatment (Figs. 4A and 4B).

### Ovulation, egg fertility and embryo survival

**Figure 5:**
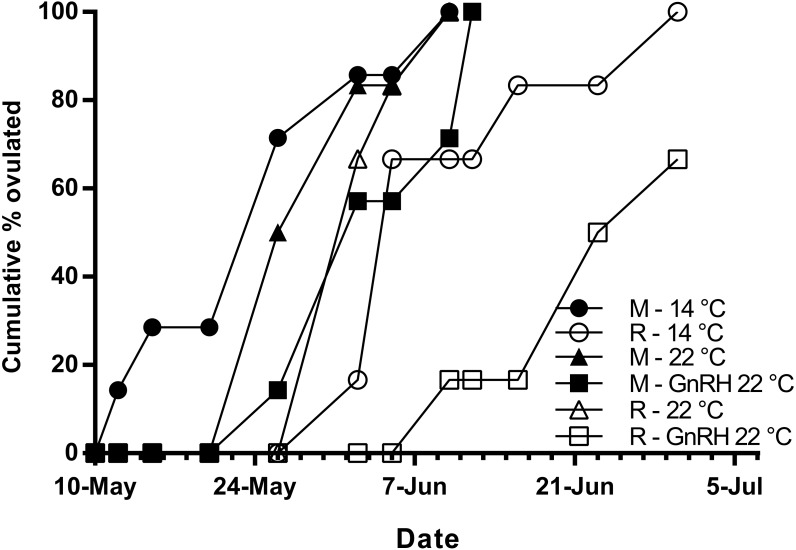
Cumulative percent of fish that had ovulated for each experimental group. M, Maiden and R, Repeat spawning fish. Other details as for Fig. 2.

**Figure 6:**
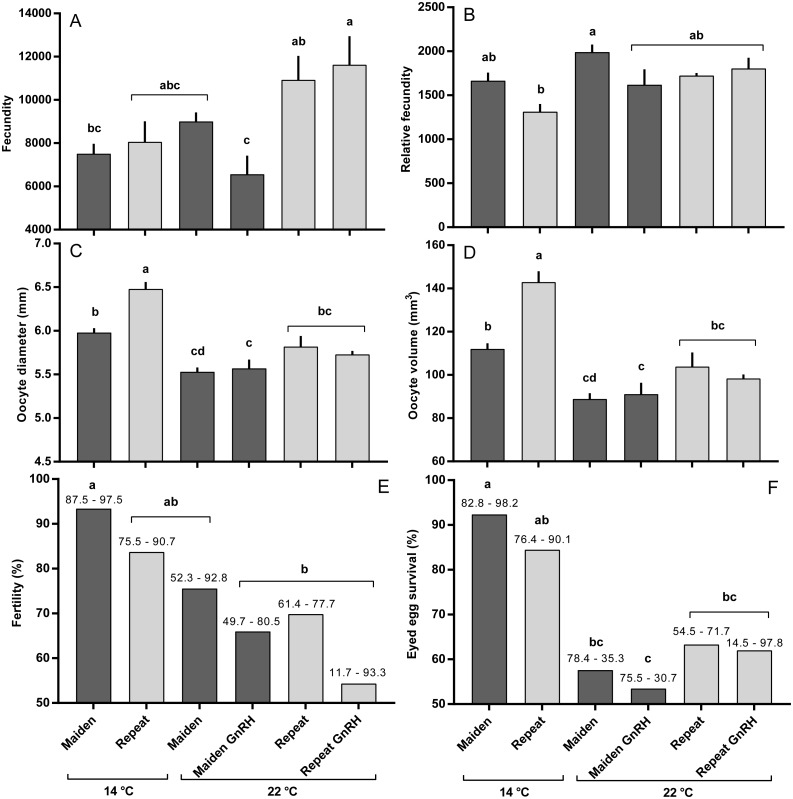
Fecundity (A), relative fecundity (B), egg size (at time of stripping, C and D), fertility (E) and survival to the eyed stage (F) for maiden and repeat spawners exposed to 14 or 22 °C with or without GnRH implantation. Values are mean + SEM (or 95% confidence limits for % data). Different superscripts denote significant differences (*p* ≤ 0.05).

Maidens held at 14 °C ovulated first, followed by a cluster of untreated and treated maidens at 22 °C, repeats at 22 °C, and then repeats at 14 °C (Fig. 5). (Repeats at 22 °C treated with GnRH showed markedly delayed ovulation relative to all other groups. Fecundity and relative fecundity at ovulation was unaffected by temperature or hormonal treatment (Figs. 6A and 6B). At 22 °C, repeat fish had slightly higher absolute, but not relative fecundity. Maiden and repeat spawning fish at 22 °C had markedly smaller post-ovulatory egg diameters and volumes (Figs. 6C and 6D) than their respective controls at 14 °C. Egg size was smaller in maiden fish than repeat fish at 14 °C, although egg size was similar between maidens and repeats at 22 °C. Fertility and eyed egg survival was higher in fish reared at 14 °C than at 22 °C; however, the reduction in embryo survival was less marked in repeats than maidens compared to the respective 14 °C control group (Figs. 6E and 6F). Treatment with GnRH did not significantly affect egg fertility or survival in maidens or repeats relative to the appropriate control at 22 °C.

### Plasma hormones and Vtg

**Figure 7:**
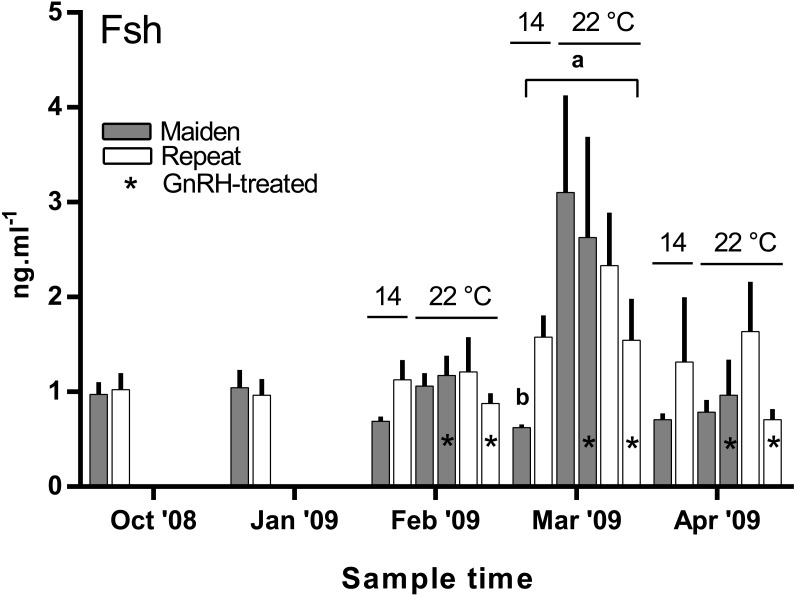
Mean + SEM (*n* = 6–7) plasma Fsh levels in maiden and repeat spawners exposed to 14 or 22 °C with or without GnRH implantation. Fsh levels below the lower limit of assay detection are displayed as 0.6 ng ml^−1^. Other details as for Fig. 2.

There was no difference in the level of plasma Fsh between groups of fish from October to February (Fig. 7). In March, plasma Fsh was elevated in treated and untreated maiden fish reared at 22 °C relative to the untreated group maintained at 14 °C, and Fsh levels were similar among both maiden groups at 22 °C. There was no significant difference among plasma Fsh levels in repeat fish in March. There was no significant difference in plasma Fsh levels between groups of fish in April. There were no differences in the mean (±SEM) plasma Lh levels between any groups of fish in March (0.91 ± 0.08 ng ml^−1^) or April (0.85 ± 0.037 ng ml^−1^) (data not shown).

**Figure 8:**
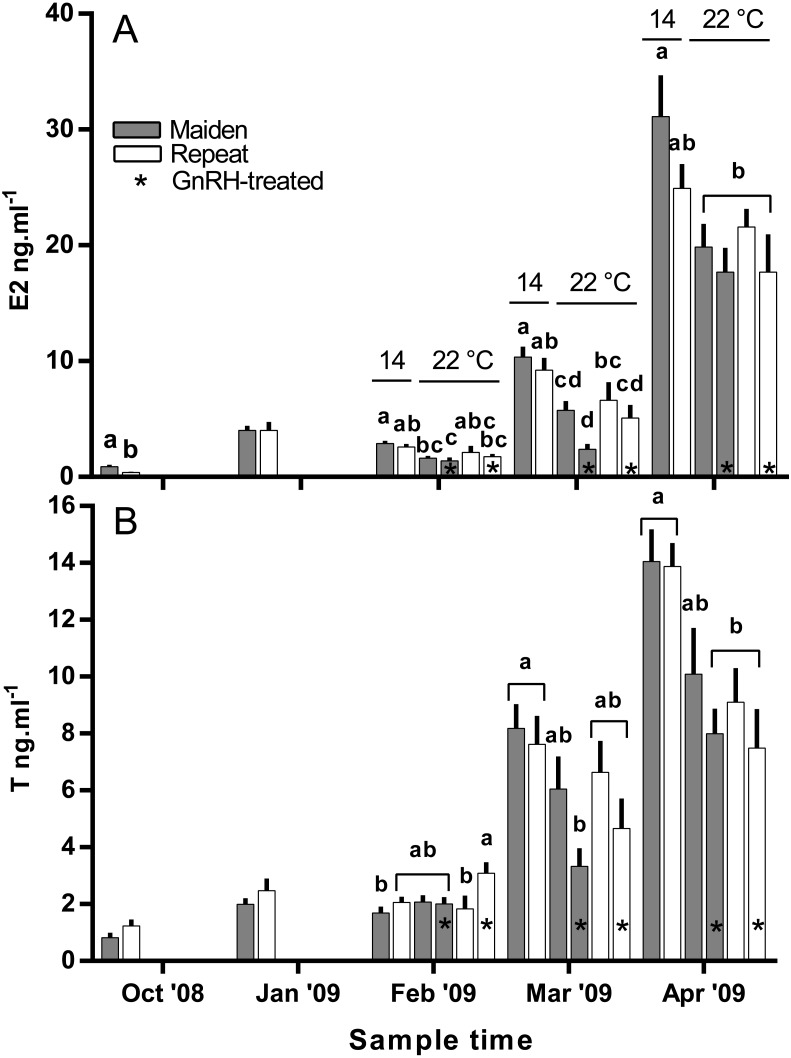
Mean + SEM (*n* = 6–7) plasma estradiol (A) and testosterone (B) levels of maiden and repeat spawners. Other details as for Fig. 2.

Plasma E2 levels were suppressed at in February in both groups of maiden, but not repeat fish reared at 22 °C relative to their respective control at 14 °C (Fig. 8A). In March, plasma E2 levels in maidens were suppressed in both groups at 22 °C and there was also suppression at 22 °C in repeats treated with GnRH. In April, all 22 °C groups were suppressed relative to maidens held at 14 °C. T levels were not different between groups in October or January, and repeat fish treated with GnRH had higher T levels than the corresponding control group at 22 °C in February (Fig. 8B). Plasma T levels were suppressed at 22 °C in maidens treated with GnRH in March and April relative to maidens at 14 °C, and were suppressed in both groups of repeats held at 22 °C in April relative to the control group at 14 °C. Mean (±SEM) plasma F levels were elevated in maidens (15.7 ± 2.0 ng ml^−1^) relative to repeats (3.3 ± 1.2 ng ml^−1^) in October, but there were no differences among groups between January and March (data not shown). In April, F was elevated in repeats at 14 °C (23.1 ± 6.4 ng ml^−1^) relative to all other groups (range 2.4–8.8 ng ml^−1^).

**Figure 9:**
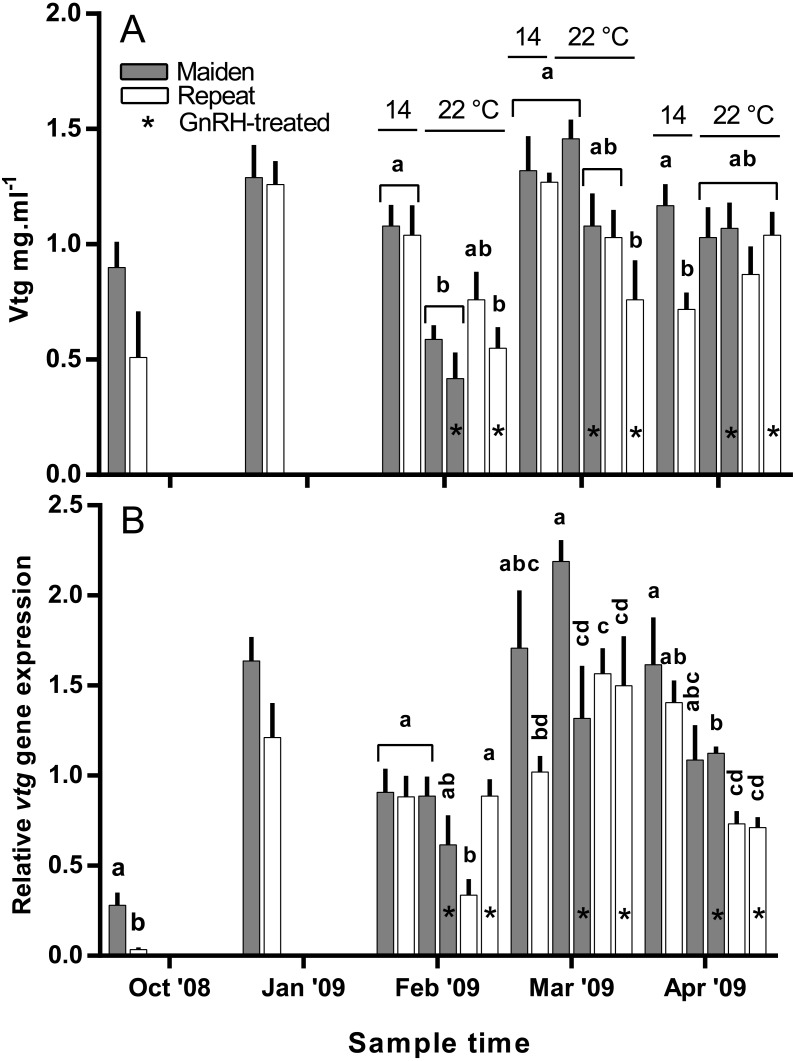
Mean + SEM (*n* = 6–7) plasma vitellogenin (A) levels, and hepatic *vtg* gene expression levels (B) of maiden and repeat spawners exposed to 14 or 22 °C with or without GnRH implantation. Gene expression was normalised to TATA binding protein. Other details as for Fig. 2.

Plasma Vtg levels increased through reproductive development and were similar among groups until February when levels were suppressed in maidens at 22 °C relative to 14 °C (Fig. 9A). In February, suppression of plasma Vtg was also observed for repeat fish treated with GnRH, and to some extent the untreated 22 °C group relative to the corresponding control at 14 °C. There was some recovery in plasma Vtg levels by March, with only repeats treated with GnRH showing significant suppression at 22 °C. In April, there were no differences between fish at 14 and 22 °C for either age class; although, maiden fish had higher levels of plasma Vtg than repeat fish at 14 °C.

### Hepatic gene expression

Hepatic levels of relative *vtg* gene expression increased markedly from November to January, and in February, *vtg* expression was suppressed in repeats at 22 °C but this effect appeared to be offset by treatment with GnRH (Fig. 9B). In maidens, *vtg* gene expression was unaffected by maintenance at 22 °C or treatment with GnRH in February. In March, *vtg* expression was suppressed in GnRH-treated maiden fish at 22 °C, compared to the 22 °C maiden control group, but not maidens at 14 °C. There were no differences among repeat GnRH-treated and control fish held at 22 °C. In April, maidens held at 22 °C and treated with GnRH showed suppression relative to maidens at 14 °C, and both groups of repeats held at 22 °C showed suppressed expression relative to repeats at 14 °C.

**Figure 10:**
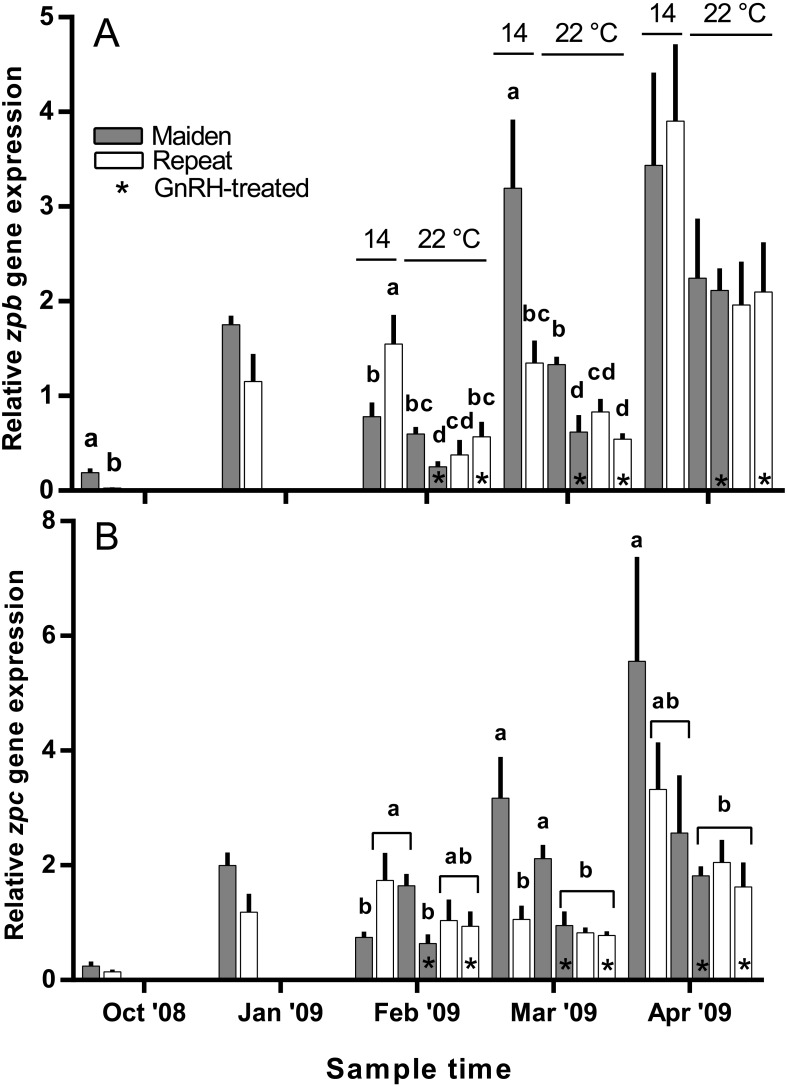
Mean + SEM (*n* = 6–7) hepatic zona pellucida B (A) and C (B) gene expression levels of maiden and repeat spawners exposed to 14 or 22 °C with or without GnRH implantation. Gene expression was normalised to TATA binding protein. Other details as for Fig. 2.

Hepatic *zpb* gene expression was down-regulated in repeat fish relative to maidens in October; although there was no difference in gene expression by January between groups (Fig. 10A). In February, *zpb* expression was significantly reduced in maiden fish at 22 °C receiving GnRH implantation relative to maidens at 14 °C, and both groups of repeats at 22 °C showed suppression relative to repeats at 14 °C. In March, *zpb* expression was suppressed in both groups of maidens held at 22 °C, and repeats at 22 °C treated with GnRH. In April, there were no significant differences between treatments even though there was a general trend towards lower gene expression at elevated temperature. In October and January, the level of *zpc* gene expression was not different between maidens and repeats (Fig. 10B). In February, *zpc* expression was higher in repeats than maidens at 14 °C, and gene expression was higher in maidens at 22 °C than at 14 °C. There was suppressed *zpc* expression in maidens held at 22 °C treated with GnRH, relative to maidens at 14 °C in March. In April, the same effect was present. There were no significant differences among fish held at 22 °C in April; however, there was a general trend towards lower gene expression levels at elevated temperature.

## Discussion

Maintenance at 22 °C with or without treatment with GnRH did not affect body weight relative to fish reared at 14 °C. CF was initially lower in repeat fish during November; however, CF was very similar between all groups from January onwards which appears to be a consistent feature for this population of *S. salar* (Pankhurst et al., 2011).

The onset of ovulation was delayed by 21 days in repeat fish relative to maidens reared at 14 °C, which is consistent with the slower rate of development in repeat fish relative to maiden fish reared at 14 °C during early vitellogenesis. In maidens, treatment with GnRH did not affect the timing of ovulation relative to the control group reared at 22 °C. In contrast, ovulation was delayed in repeat spawning fish treated with GnRH relative to the control group at 22 °C; however, this was not associated with a further reduction in egg fertility and embryo survival. While other factors may be involved, delays in ovulation at high temperature in salmonids are thought to be caused by dopamine mediated inhibition of Lh secretion (Gillet, Breton & Mikolajczyk, 1996), in combination with impaired ovarian steroidogenesis, and thus lower circulating levels of maturation inducing steroid (17,20β-dihydroxy-4-pregnen-3-one, MIS) in the lead up to ovulation (Anderson et al., 2017; King & Pankhurst, 2004b). Treatment with GnRH had no affect on plasma Lh levels; however, due to the timing of sampling relative to the time of ovulation, the sampling schedule was unlikely to have detected the preovulatory rise in plasma Lh associated with FOM described by Breton, Govoroun & Mikolajczyk (1998). Furthermore, it is unlikely that GnRH implantation directly affected the events associated with FOM and ovulation, as the Ovaplant™ pellet has a sustained release profile of approximately 24 days total at 10 °C, and the second implantation occurred approximately 3 months before ovulation commenced in untreated repeats at 22 °C.

Exposure to elevated temperature alone or in combination with prolonged GnRH-treatment did not affect GSI, relative fecundity or fecundity for maiden or repeat spawning fish during oocyte development or at ovulation. GnRH-treatment also did not affect follicle diameter during vitellogenesis, and in April all GnRH-treated and untreated groups maintained at 22 °C displayed oocyte diameters that were smaller than those from fish maintained at 14 °C. Plasma Vtg levels were lower in GnRH-treated maiden and repeat fish maintained at 22 °C in February, and repeat fish only in March, relative to the corresponding control group at 14 °C. Since treatment with GnRH failed to stimulate the production of Vtg at 22 °C in maidens and repeats, Vtg uptake by oocytes was presumably reduced and follicle diameter consequently smaller in GnRH-treated fish compared to the respective control group at 14 °C. At the time of stripping, all maidens and repeats reared at 22 °C with or without hormonal treatment had lower egg diameters and volumes compared to the corresponding control group at 14 °C, which is consistent with the smaller follicle diameters observed for these groups during late vitellogenesis. Egg fertility and survival was generally reduced in maiden and repeat fish at 22 °C relative to the respective control at 14 °C, and consistent with our previous work, repeats appeared to be slightly more resilient to the effects of thermal challenge (Pankhurst et al., 2011). GnRH therapy during vitellogenesis did not help to maintain egg quality in maiden or repeat fish reared at 22 °C, with the lowest fertility and eyed egg survival rates recorded for GnRH-treated fish.

In the present study, GnRH-therapy during vitellogenesis failed to elevate plasma Fsh levels in maiden and repeat fish maintained at 22 °C above those recorded for the corresponding untreated group at 22 °C. In salmonids, GnRH-treatment is able to stimulate Fsh production and secretion in primary pituitary cell cultures (Dickey & Swanson, 2000), although pituitary responsiveness to GnRH stimulation is dependent on the stage of reproductive development (Ando et al., 2004). In *O. mykiss*, treatment with a dopamine agonist inhibited the (GnRH-induced) release of Fsh from the pituitary during late vitellogenesis (Vacher et al., 2000), and in *S. alpinus*, it is thought that dopamine is involved in the thermal inhibition of ovulation (Gillet & Breton, 2009). In the latter study, treatment with GnRH in combination with a dopamine antagonist was more effective at inducing ovulation than treatment with GnRH alone at high temperature (Gillet & Breton, 2009). Therefore, it is possible that GnRH-treatment during vitellogenesis could be more effective at 22 °C if administered in combination with a dopamine antagonist. However, it should be noted that plasma Fsh levels were unchanged as a result of thermal challenge in untreated repeat fish, and in maiden fish, plasma Fsh levels where higher at 22 °C relative to the control group at 14 °C. Sex steroids such as E2 are known regulators of pituitary hormones (Pankhurst, 2008); for example in *O. kisutch*, E2 has been shown to down-regulate pituitary *fshβ* gene expression during secondary oocyte growth (Dickey & Swanson, 1998). In the present study, plasma E2 was suppressed in maidens but not repeats maintained at 22 °C in February and March. Therefore a reduction in negative feedback by E2 on the pituitary may have resulted in an increase in plasma Fsh in maidens maintained at 22 °C as suggested previously for this stock of *S. salar* (Anderson & Elizur, 2012).

There was no evidence to suggest that treatment with GnRH at 22 °C compensated for the negative effects of elevated temperature on the production of T in the present study, other than in February in repeat spawning fish when plasma T was higher in GnRH-treated fish than in the corresponding control group at 22 °C. The stimulatory effect of GnRH on plasma T for repeat fish at that sample did not correspond to an increase in plasma E2 for the same or following months. In a previous study on *S. salar*, treatment with GnRH increased plasma T levels relative to sham treated fish during vitellogenic development (Crim, Glebe & Scott, 1986). This implies that any stimulatory effect of GnRH-treatment achieved in the present study was not significant enough to compensate for impairment of endocrine function at 22 °C.

In the present study, plasma E2 levels were generally lower in maiden and repeat spawning fish treated with GnRH at 22 °C compared to the corresponding control group at 14 °C. This is presumably due to the inability of GnRH to stimulate the production of T, and the down-regulation of P450 aromatase A (*cyp19a1a*) typically observed in this stock of fish at 22 °C (Anderson & Elizur, 2012). In pink salmon (*Oncorhynchus gorbuscha*), prolonged treatment with GnRH effectively elevated plasma E2 levels during reproductive development only when administrated in combination with T in fish reared at between 8.9–12.3 °C (Crossin et al., 2010). However, co-treatment of fish with GnRH and T may not promote vitellogenic development at higher temperatures in *S. salar* if there is thermal inhibition at the level of gonadal *cyp19a1a*. In addition, thermal impairment of steroidogenesis upstream of T may have played a role, as the gene expression of several steroidogenic enzymes are downregulated in *S. salar* at 22 °C (Anderson et al., 2017; Anderson et al., 2012).

Plasma F was elevated in maiden fish relative to repeats in October, which is likely to be a reflection of the crowding method used to capture this group of fish early in the experiment (Pankhurst et al., 2011). Between January and March plasma F levels in all fish were lower than those typically observed in stressed *S. salar* (Thomas, Pankhurst & Bremner, 1999) which indicates that treatment with GnRH and exposure to high temperature was not perceived as stressful. In April, plasma F was elevated in repeat fish reared at 14 °C relative to all other groups. However, the concentration of plasma F in repeat fish maintained at 14 °C (23.14 ± 6.4 ng ml^−1^) was similar to the level observed in unstressed *S. salar* and *O. mykiss* (∼15 ng ml^−1^ for each study) (Pankhurst & Van Der Kraak, 2000; Thomas, Pankhurst & Bremner, 1999), and it is therefore unlikely that F levels in repeat fish were high enough to significantly impact reproductive development in the present study.

Treatment with GnRH at 22 °C had no effect on *vtg* gene expression in maiden or repeat fish, except in February where *vtg* gene expression was up-regulated in repeat fish after GnRH-treatment relative to untreated repeats at 22 °C. However, the stimulatory effect of GnRH-treatment observed for repeat fish in February did not cause a subsequent increase in plasma Vtg. Furthermore, treatment with GnRH at 22 °C had no effect on plasma levels of Vtg in maiden and repeat spawning female *S. salar*; except in March when plasma Vtg was significantly suppressed in GnRH-treated repeat fish at 22 °C relative to the control at 14 °C. This is not surprising since Vtg production is regulated by E2 (Lubzens et al., 2010), and GnRH-treatment failed to elevate plasma E2 or *vtg* gene expression levels at 22 °C.

Treatment with GnRH did not help to maintain *zpb* gene expression levels at 22 °C in repeat fish at any time during reproductive development. For maidens, treatment with GnRH had an inhibitory effect on *zpb* gene expression in February and March, and no effect in April. Similarly, *zpc* gene expression was down-regulated in response to GnRH-treatment in maiden but not repeat fish in February and March, and there was no significant effect on any fish in April. This suggests that female *S. salar* respond differently to GnRH-treatment on the basis of stock age in terms of Zp transcription, although the molecular basis for this phenomenon is unclear given the fact that plasma E2 levels were similar between GnRH-treated maidens and repeats throughout the study.

The present study was undertaken to determine whether reductions in egg quality caused by thermally induced endocrine impairment could be compensated for by prolonged treatment with GnRH during vitellogenic development. It is clear that treatment with GnRH alone is not an effective strategy for enhancing the endogenous production of plasma E2 and maintaining egg quality at elevated temperature. While maiden and repeat fish responded in a similar way to treatment with GnRH for almost all of the parameters measured, we have provided evidence suggesting that *S. salar* broodstock respond differently to hormonal manipulation in terms of *zp* gene expression although the molecular basis for this effect is unclear. As GnRH-treatment was not able to overcome the thermal inhibition of multiple endocrine events upstream of E2 production, the direct administration of E2 warrants investigation. Direct administration of E2 may maintain egg quality at high temperature in female *S. salar* broodstock by surpassing upstream endocrine impairments, and stimulating the down-stream synthesis of Vtg and Zps.

##  Supplemental Information

10.7717/peerj.3898/supp-1 Supplemental Information 1 All raw data for GnRHa-treatment study

10.7717/peerj.3898/supp-2 Table S1 Candidate reference genes with the highest stability as determined by different algorithms, at each sampling date, and for sampling dates combined The numbers in each cell represent the stability value calculated by each algorithm.

## Additional Information and Declarations

### Data Availability

The following information was supplied regarding data availability: The raw data has been provided as a Supplemental File.
