# Supplementary material for: Effects of GnRHa treatment during vitellogenesis on the reproductive physiology of thermally challenged female Atlantic salmon (Salmo salar)
Source: PeerJ. 2017 Oct 20;5:e3898. doi: 10.7717/peerj.3898 (PMC5652270; doi:10.7717/peerj.3898)
Supplement: Table S1 — The numbers in each cell represent the stability value calculated by each algorithm. [file peerj-05-3898-s002.docx]

**Figure S1.** Candidate reference genes with the highest stability as determined by different algorithms, at each sampling date, and for sampling dates combined. The numbers in each cell represent the stability value calculated by each algorithm.

| **Sample date** | **Comprehensive** | **Genorm** | **BestKeeper** | **NormFinder** |
| --- | --- | --- | --- | --- |
| Nov-08 | Hprt1 1.19 | Hprt1/Beta 0.81 | TATA 0.578 | TATA 0.30 |
| Jan-09 | EF1α 1.32 | Ef1α /Hprt1 2.03 | TATA 0.76 | Ef1α 1.01 |
| Feb-09 | TATA 1.19 | TATA/Hprt1 0.89 | TATA 1.023 | TATA 0.33 |
| Mar-09 | TATA 1.56 | TATA/Hprt1 0.95 | TATA 0.82 | TATA 1.24 |
| Apr-09 | TATA 1.0 | TATA/Hprt1 0.57 | TATA 1.12 | TATA 0.28 |
| All data | TATA 1.41 | TATA/Ef1α 2.462 | TATA 1.05 | Hprt1 1.12 |
